# Galantamine anti-colitic effect: Role of alpha-7 nicotinic acetylcholine receptor in modulating Jak/STAT3, NF-κB/HMGB1/RAGE and *p*-AKT/Bcl-2 pathways

**DOI:** 10.1038/s41598-018-23359-6

**Published:** 2018-03-23

**Authors:** Shakeeb A. Wazea, Walaa Wadie, Ashraf K. Bahgat, Hanan S. El-Abhar

**Affiliations:** 1 Department of Pharmacology & Toxicology, Faculty of Pharmacy, Cairo University, Cairo, Egypt; 2 Department of Pharmacology & Toxicology, Faculty of Pharmacy, Future University in Egypt, Cairo, Egypt

## Abstract

Vagal stimulation controls systemic inflammation and modulates the immune response in different inflammatory conditions, including inflammatory bowel diseases (IBD). The released acetylcholine binds to alpha-7 nicotinic acetylcholine receptor (α7 nAChR) to suppress pro-inflammatory cytokines. This provides a new range of potential therapeutic approaches for controlling inflammatory responses. The present study aimed to assess whether galantamine (Galan) anti-inflammatory action involves α7 nAChR in a 2,4,6-trinitrobenzene sulfonic acid (TNBS) model of colitis and to estimate its possible molecular pathways. Rats were assigned into normal, TNBS, sulfasalazine (Sulfz), Galan treated (10 mg/kg), methyllycaconitine (MLA; 5.6 mg/kg), and MLA + Galan groups. Drugs were administered orally once per day (11 days) and colitis was induced on the 8^th^ day. Galan reduced the TNBS-induced ulceration, colon mass index, colonic MDA, neutrophils adhesion and infiltration (ICAM-1/MPO), inflammatory mediators (NF-κB, TNF-α, HMGB1, and RAGE), while increased the anti-apoptotic pathway (*p*-Akt/Bcl-2). Mechanistic study revealed that Galan increased the anti-inflammatory cytokine IL-10, phosphorylated Jak2, while reduced the inflammation controller SOCS3. However, combining MLA with Galan abrogated the beneficial anti-inflammatory/anti-apoptotic signals. The results of the present study indicate that Galan anti-inflammatory/-apoptotic/ -oxidant effects originate from the stimulation of the peripheral α7 nAChR, with the involvement of the Jak2/SOCS3 signaling pathway.

## Introduction

According to the cytokine theory of disease, balanced low levels of cytokines are required to maintain physiological homeostasis, while the overproduction of some cytokines associates various diseases^1^, including inflammatory bowel disease (IBD)^2,3^. In particular, the imbalance between pro- and anti-inflammatory cytokines, which ensues in IBD, hinders the resolution of inflammation and instead leads to disease propagation and tissue destruction^4^.

Hence, inhibiting the production or neutralization of these pro-inflammatory cytokines in inflamed intestinal mucosa of IBD is the goal of treating the disease. Besides the conventional treatments; *viz*., aminosalicylates and corticosteroids, anti-cytokine agents have been considered recently for IBD therapy^5,6^. Additionally, several studies verified the role of the vagus nerve in controlling the production of several pro-inflammatory cytokines to control systemic inflammation in different inflammatory conditions through what is known as the “cholinergic anti-inflammatory pathway”^7,8^. The latter is a mechanism by which local inflammation activates the afferent (sensory) fibers of the vagus nerve to signal the brain to trigger an anti-inflammatory response through firing of the efferent vagus nerve^9^.

Acetylcholine (ACh), released by stimulation of the vagus nerve, binds to the α7 nicotinic acetylcholine receptor (α7 nAChR) expressed on macrophages to suppress pro-inflammatory cytokine production. This anti-inflammatory pathway can be manipulated with cholinergic agonists, such as nicotine or with electrical stimulation of the vagus nerve^10^. Hence, activation of this receptor can provide a new scope of possible therapeutic approach for controlling host inflammatory responses during inflammatory conditions. Accordingly, two selective α7 nAChR agonists in addition to nicotine itself were examined in a dextran sulfate sodium (DSS)-induced model of colitis in mice. Contrary to expectation, the study revealed that both agonists exacerbated the DSS effects, while nicotine failed to affect disease parameters although it reduced colonic cytokine production^11^.

Cholinesterase inhibitors (ChEIs) that increase local concentrations of ACh and penetrate the central nervous system (CNS) may be more effective and less toxic than nicotinic agonists in treating colitis. Hence, in an experimental model of colitis in rats, both neostigmine and physostigmine reduced the colonic macroscopic damage and neutrophils infiltration, with the latter showing a better effect, possibly due to its ability to penetrate the CNS. Therefore, it was concluded that stimulation of the central cholinergic receptors contribute more to the anti-inflammatory effect of physostigmine^12^. In addition, rivastigmine reduced colitis by increasing ACh that activates α7 nAChRs on circulating macrophages and brainstem neurons^13^.

Galantamine (Galan), a reversible competitive ChEI that crosses the blood brain barrier, increases brain cholinergic network activity and is widely used in the treatment of Alzheimer’s disease^14^ by increasing the ACh level and by its unique powerful binding capacity to the allosteric site on α7 nAChR^15,16^. Galan activates the efferent vagus nerve^17^ and its anti-inflammatory character has been associated with the activation of brain muscarinic acetylcholine receptor M1^18^ to trigger the cholinergic anti-inflammatory pathway^8,19^. The latter co-workers reported that Galan lessened the severity of mucosal inflammation depending entirely on the integrity of vagus and splenic nerves^8^.

In the current study, we aimed to investigate the possible interpretation of the peripheral α7 nAChRs in the anti-colitic anti-inflammatory effect of Galan, apart from its central M1 receptor activation, and to explore some of the possible mechanisms/molecular pathways involved using a TNBS-induced colitis model in rats. Sulfasalazine (Sulfz), one of the classical standard treatments of colitis, was used as the reference drug.

## Material and Methods

### Animals

Adult male Wistar rats, weighing 150–200 g, (Modern Veterinary Office for Laboratory Animals, Cairo, Egypt) were acclimatized for 2 weeks before experimentation. Animals were kept under controlled environmental conditions of, constant temperature (25 ± 2 °C), humidity (60 ± 10%), and a 12/12-h light/dark cycle. Rats were allowed free access to standard chow pellets and water *ad libitum*. The study complies with the Guide for the Care and Use of Laboratory Animals published by the US National Institutes of Health (NIH Publication no. 85–23, revised 2011) and was approved by the Research Ethics Committee for Animal Experimentation at the Faculty of Pharmacy, Cairo University, Cairo, Egypt under the permit number (PT 1088; 30/4/2013).

### Induction of colitis

Colitis was induced by TNBS (Fluka, St. Gallen, Switzerland) applying essentially the method described by Morris, *et al*.^20^. Rats were lightly anesthetized and TNBS (0.25 ml; 50% ethanol v/v) was administered into the colon lumen through a polyethylene catheter inserted via the rectum to 8 cm from the anus.

### Experimental design and drug treatment

Two sets of experiments were carried out, one with a view to select the protective dose of Galan against TNBS-induced colitis and the second to investigate the possible mechanisms involved.

#### Assessment of the possible protective effect of Galan against TNBS-induced colitis

In the first set, rats (n = 48) were equally assigned into 2 control groups (normal and TNBS), 3 groups receiving different doses of Galan (2.5, 5 and 10 mg/kg), and one Sulfz treated group (300 mg/kg). Drugs were administered orally (intragastric) once per day throughout the experimental period (11 days). On day 8, colitis was induced by intra-colonic instillation of TNBS in all groups except normal control group, which received normal saline instead. Animals were weighed immediately before colitis induction and just before autopsy to determine whether TNBS had an effect on body weight (BW). Sulfz (Colosalazine^®^, Alexandria Company for Pharmaceuticals & Chemical Industries, Alexandria, Egypt) and Galan (Reminyl^®^, Janssen-Cilag, Beerse, Belgium) were purchased in their commercial preparation forms. Sulfz was used as a suspension in 1% methylcellulose. The concentration of all drugs was adjusted, so that 1 ml of solution/ suspension was given per 200 g of rat weight. Twenty four hours after the last treatment dose, rats were sacrificed by an overdose of thiopental and the distal 8 cm of the colon was excised, opened longitudinally, cleaned, and weighed. Mucosal damage was assessed by measuring the ulcerative area (cm^2^) and the ratio of colon weight (mg) to total BW (g) was taken as the colon mass index (CMI) that was used as a measure of colonic edema degree and severity of inflammation^21^. The colon was then homogenized in ice-cold normal saline to obtain a 10% (w/v) homogenate that was divided into three aliquots. One aliquot was mixed with an equal volume of phosphate buffer (100 mM; pH 6) containing 1% hexadecyltrimethyl-ammonium bromide (HTAB). The solution was sonicated for 10 seconds, then centrifuged at 10,000 rpm for 15 min at 4 °C. The supernatant was used for spectrophotometric estimation of myeloperoxidase (MPO) activity according to the methods of Bradley *et al*.^22^. The second and third aliquots were used for the estimation of reduced glutathione (GSH) and malondialdehyde (MDA) using the corresponding spectrophotometric kits (Biodiagnostic Co., Giza, Egypt).

Based on the results of this experiment, the high dose level of Galan 10 mg/kg (Galan_10_) was selected for further experimentation. As an indirect confirmation for the ability of Galan_10_ to increase ACh output in TNBS-induced colitis, the above experiment was again repeated with only three main groups; viz., normal control, TNBS control, and Galan_10_ (n = 6). Twenty four hours after the last dose of treatment, animals were anaesthetized with thiopental and blood samples were collected from the femoral vein to prepare plasma samples that were used for the determination of ACh level (MyBioSource ELISA kit, CA, USA).

#### Investigation of the possible mechanism of the protective effect of Galan (10 mg/kg)

In the second set of experiment, rats (n = 36) were allocated equally into 2 control groups (normal and TNBS), one reference group (Sulfz), one treated with blocker alone (methylcaconitine citrate [MLA], 5.6 mg/kg, i.p; Tocris Bioscience, Bristol, UK) and 2 treated with Galan_10_, with and without MLA. The blocker was administered 15 min before Galan_10_ and was dissolved immediately before injection in saline solution^23,24^. Animals were subjected to TNBS-induced colitis as mentioned before, except for the normal control group.

After sacrifice, the ulcerative area was measured and then the excised portion of colon was homogenized and divided into aliquots. In one aliquot, the colonic MPO activity was evaluated as mentioned above.

Using the corresponding enzyme-linked immunosorbent assay (ELISA) kits, the colon homogenate was used to assess the following parameters. Phosphorylated Janus kinase 2 [Tyr221] (*p*-Jak2; Jentaur, Paris, France), suppressor of cytokine 3 signaling (SOCS3; Cluod-clone Corp, Houston, USA), phosphorylated protein kinase B [pS473] (*p*-Akt; DRG, New Jersey, USA), B-cell lymphoma 2 (Bcl-2; Cusabio, Wuhan, China), and high mobility group box 1 (HMGB1; IBL international, Hamburg, Germany). Phosphorylated nuclear factor kappa B-p65 [S536] (*p*-NF-κBp65) and intercellular adhesion molecule 1 (ICAM-1) were assessed using ELISA kits purchased from EIAab (Wuhan, China), whereas kits for tumor necrosis factor alpha (TNF-α), interleukin 10 (IL-10), and receptor for advanced glycation end products (RAGE) were procured from RayBiotech Inc. (Norcross, USA).

### Histological Examination

The colonic segments from 2 representative animals/group were fixed in 10% (v/v) formalin and preserved for histological examination. Transverse sections (4–6 μm) were prepared from paraffin-embedded colon segments. The sections were stained with hematoxylin and eosin (H & E), and were examined under a light microscope.

### Statistical analysis

Values are presented as means ± S.E.M. Comparisons between means were carried out using one way analysis of variance (ANOVA), followed by Tukey’s multiple comparisons test. Differences were considered significant at *P* < 0.05. GraphPad Prism 5 (San Diego, CA, USA) was used to carry out the statistical tests.

## Results

### Preliminary study to select the suitable dose of Galan

**Figure 1:**
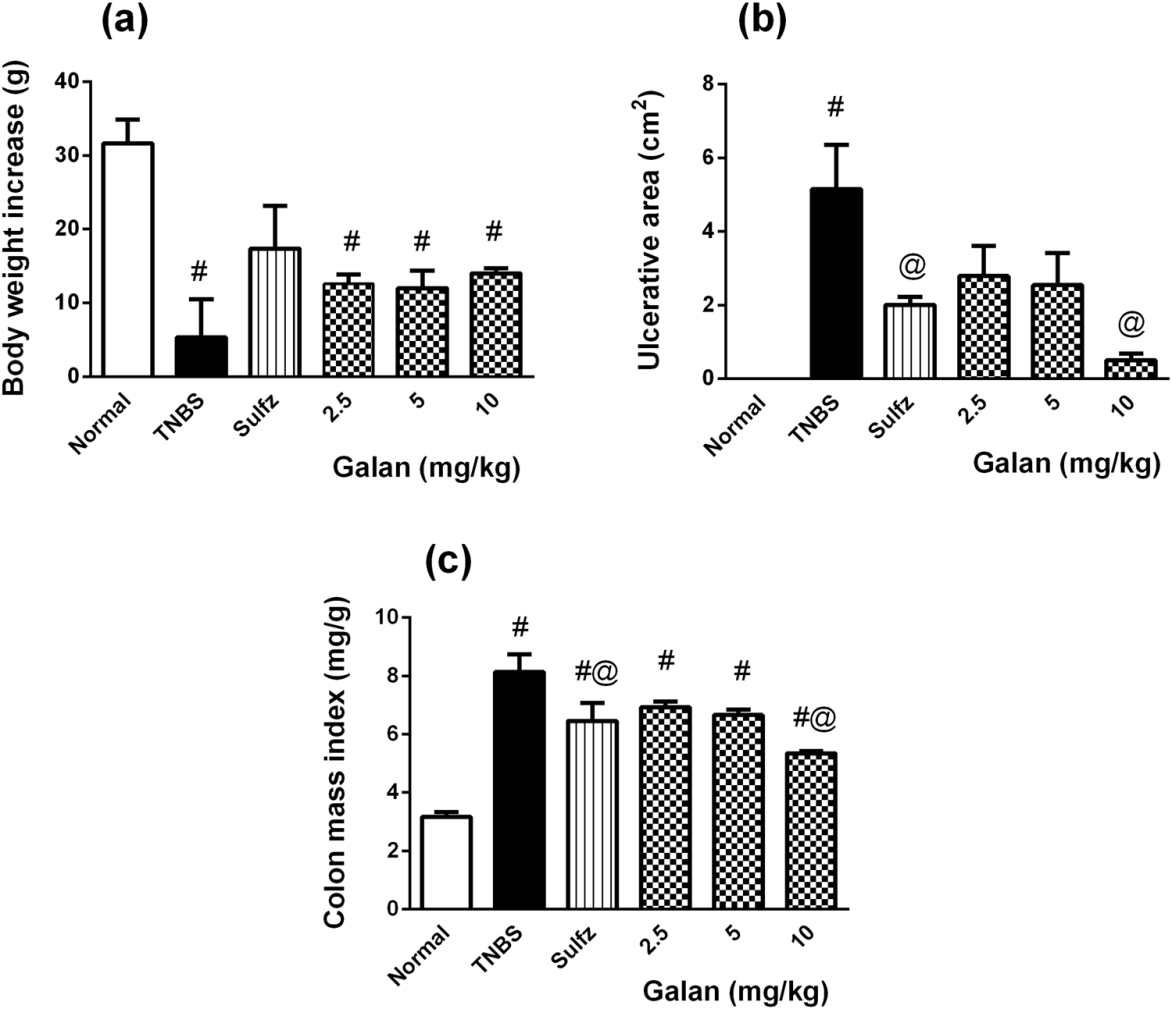
Effect of pretreatment with galantamine (Galan) on (**a**) body weight (**b**) ulcerative area (**c**) colon mass index in rats with TNBS-induced colitis. Data are expressed as means ± S.E.M. Statistical analysis was performed using one-way ANOVA followed by Tukey’s Multiple Comparison; as compared to normal control (#) and TNBS (@).

As depicted in Fig. 1a, untreated colitic rats showed a sharp decline in their BW, while pre-treatment with Sulfz prevented this decline. Galan, at all dose levels, tended to improve the rate of weight gain of animals, but with no statistical significance. Galan, in a dose dependent manner, hindered the TNBS-induced extensive ulceration, with the high dose level (Galan_10_) showing a comparable effect to that mediated by Sulfz (Fig. 1b). CMI was reduced by Galan_10_ and Sulfz as compared to that of TNBS controls, but it was still much higher than that of normal control animals (Fig. 1c).

**Figure 2:**
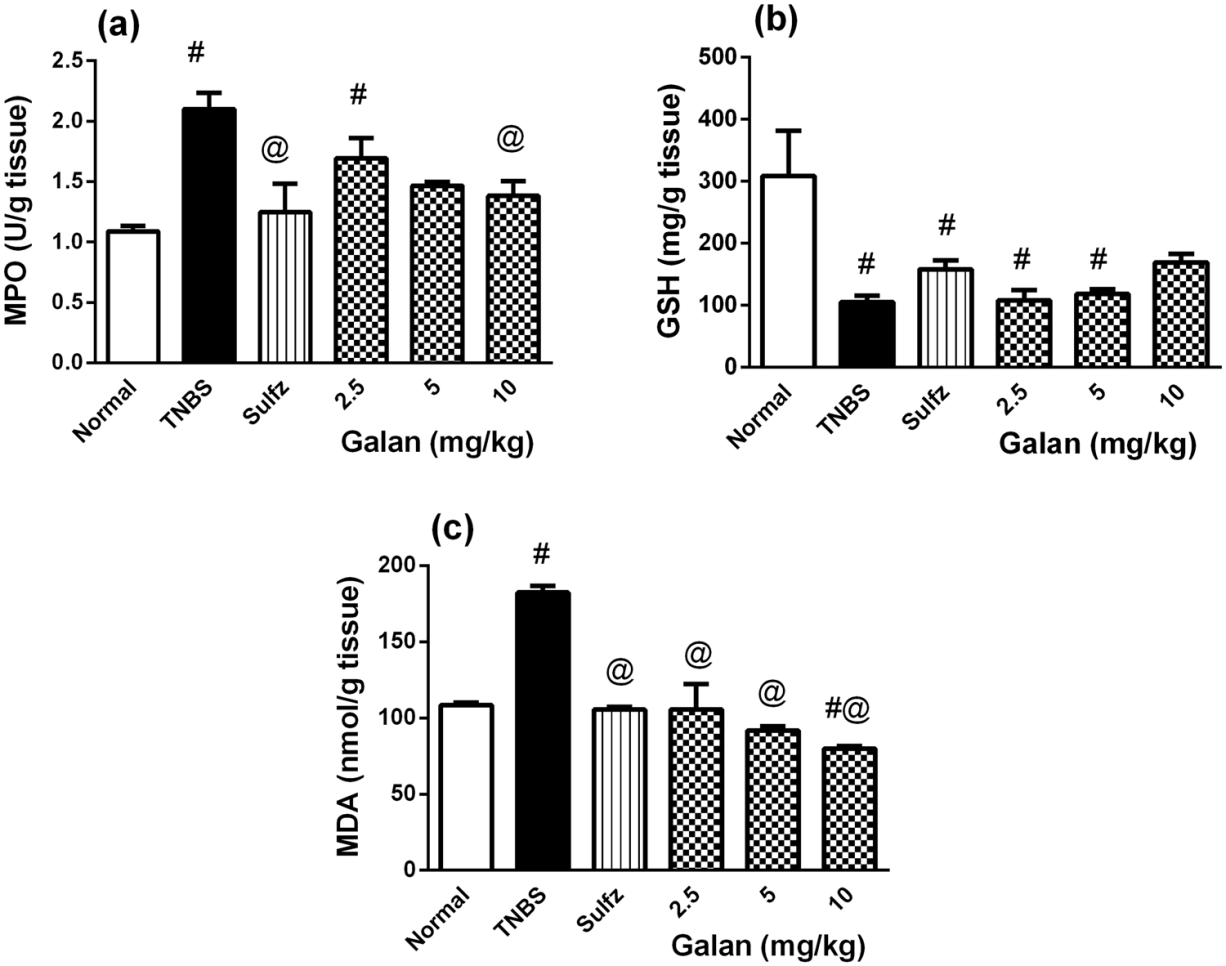
Effect of pretreatment with galantamine (Galan) on colonic (**a**) myeloperoxidase (MPO) activity, (**b**) glutathione (GSH) content, and (**c**) malondialdehyde (MDA) content in rats with TNBS-induced colitis. Data are expressed as means ± S.E.M. Statistical analysis was performed using one-way ANOVA followed by Tukey’s Multiple Comparison; as compared to normal control (#) and TNBS (@).

Intracolonic instillation of TNBS caused 2 folds rise in colonic MPO activity, an effect that was encumbered to different extents by the 3 dose levels of Galan; again the high dose offered the best effect, which was comparable to that of Sulfz (Fig. 2a). Intra-colonic administration of TNBS induced oxidative stress that depleted colonic GSH content (Fig. 2b), but almost doubled colonic MDA content (Fig. 2c). Galan and Sulfz abated the TNBS effect on MDA, however, they failed to impede GSH depletion provoked by TNBS.

### Effect of Galan_10_ on plasma level of ACh in TNBS-induced colitis

**Figure 3:**
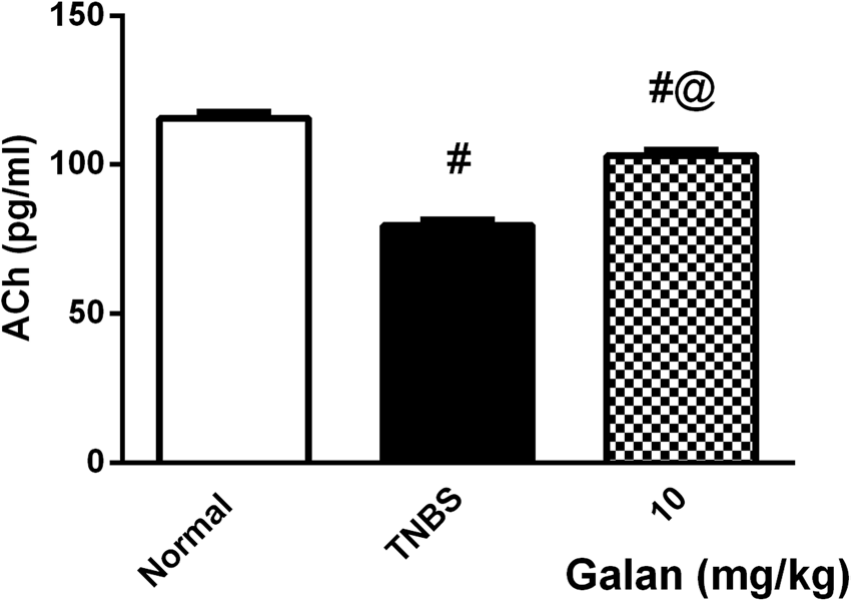
Effect of pretreatment with Galantamine (10 mg/kg; Galan_10_) on plasma level of acetylcholine (ACh) in rats with TNBS-induced colitis. Data are expressed as means ± S.E.M. Statistical analysis was performed using one-way ANOVA followed by Tukey’s Multiple Comparison; as compared to normal control (#) and TNBS (@).

As depicted in Fig. 3, TNBS caused a drop in plasma ACh level, an effect that was hindered by the use of Galan_10_.

### Effect of Galan_10_ alone and with MLA on ulcerative area, ICAM-1and MPO in TNBS-induced colitis

**Figure 4:**
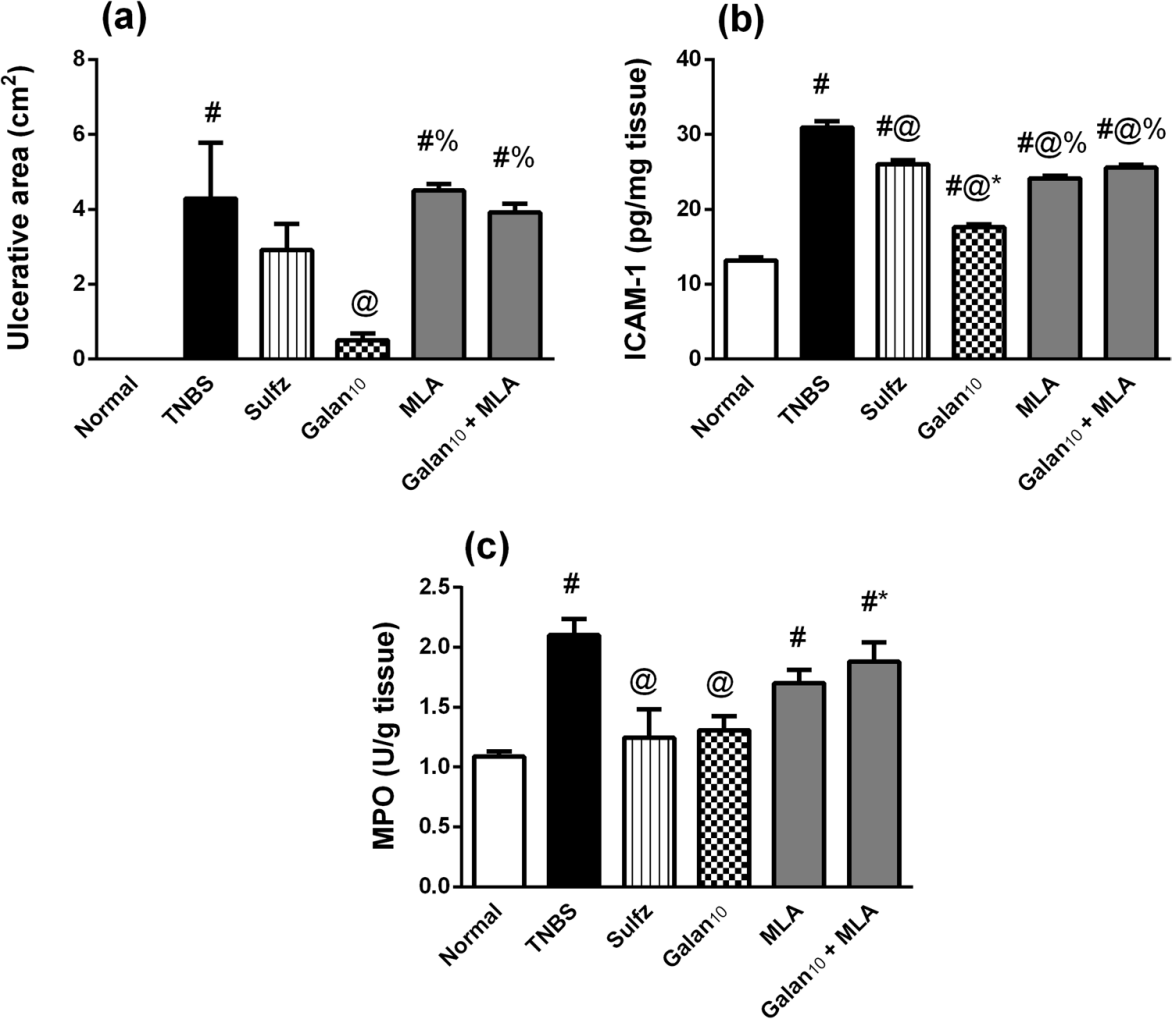
Effect of pretreatment with Galantamine (10 mg/kg; Galan_10_) alone and with methylcaconitine citrate (MLA, 5.6 mg/kg) on (**a**) ulcerative area, (**b**) colonic intracellular adhesion molecule (ICAM)-1 content and (**c**) colonic myeloperoxidase (MPO) activity in rats with TNBS-induced colitis. Data are expressed as means ± S.E.M. Statistical analysis was performed using one-way ANOVA followed by Tukey’s Multiple Comparison; as compared to normal control (#),TNBS (@), Sulfz (*), and Galan_10_ (%).

As mentioned above, Fig. 4 shows that Galan_10_ substantially prevented (a) the extensive TNBS-induced ulceration, while Sulfz produced a lesser protective effect. There was a parallel increase in (b) the adhesion molecule ICAM-1 and (c) MPO activity in the colitic group. Pretreatment with Galan almost halved ICAM-1 content, while Sulfz showed a less potent effect (15.7%); additionally, both drugs normalized MPO activity. As a proof on the involvement of α7 nAChR, the protective effects of Galan on the previous parameters were abrogated when Galan was combined with MLA, whereas MLA alone did not produce any significant change from the TNBS control group.

### Effect of Galan_10_ alone and with MLA on p-NF-κB, TNF-α, HMGB1, RAGE and IL-10 in TNBS-induced colitis

**Figure 5:**
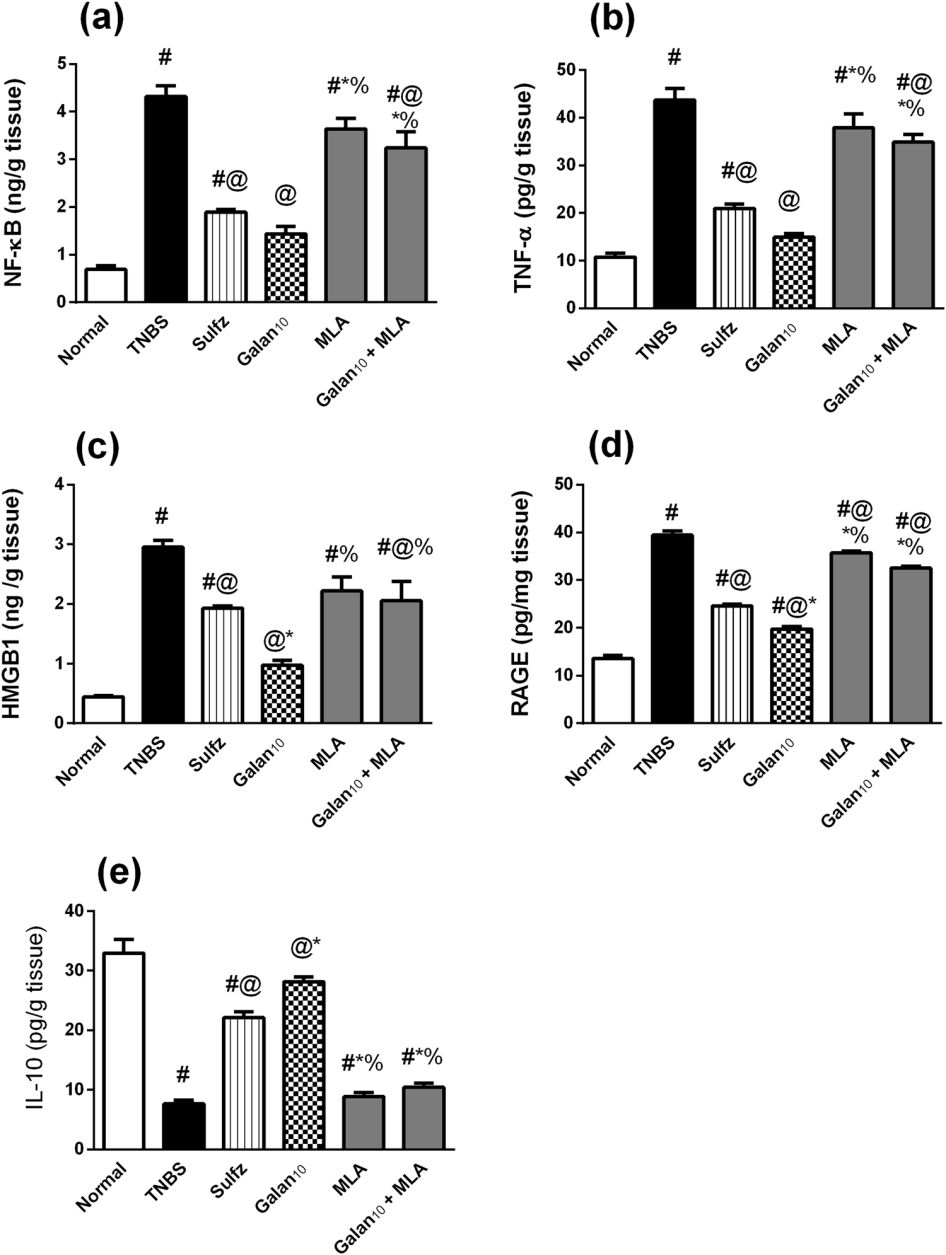
Effect of pretreatment with Galantamine (10 mg/kg; Galan_10_) alone and with methylcaconitine citrate (MLA, 5.6 mg/kg) on colonic content of (**a**) p-NF-κB, (**b**) TNF-α, (**c)** HMGB-1, (**d**) RAGE and (**e**) IL-10 in rats with TNBS-induced colitis. Data are expressed as means ± S.E.M. Statistical analysis was performed using one-way ANOVA followed by Tukey’s Multiple Comparison; as compared to normal control (#),TNBS (@), Sulfz (*), and Galan_10_ (%).

As presented in Fig. 5, TNBS boosted the colonic contents of (a) *p*-NF-κB, (b) TNF-α, (c) HMGB1, and (d) RAGE, but depleted the anti-inflammatory cytokine (e) IL-10. Treatment with Galan, however, curbed these elevations; these effects override that of Sulfz. The anti-inflammatory capacity of Galan was completely blocked upon using MLA with Galan, while MLA alone showed no alterations as compared to TNBS.

### Effect of Galan_10_ alone and with MLA on p-Jak2/SOCS3 signaling pathway in TNBS-induced colitis

**Figure 6:**
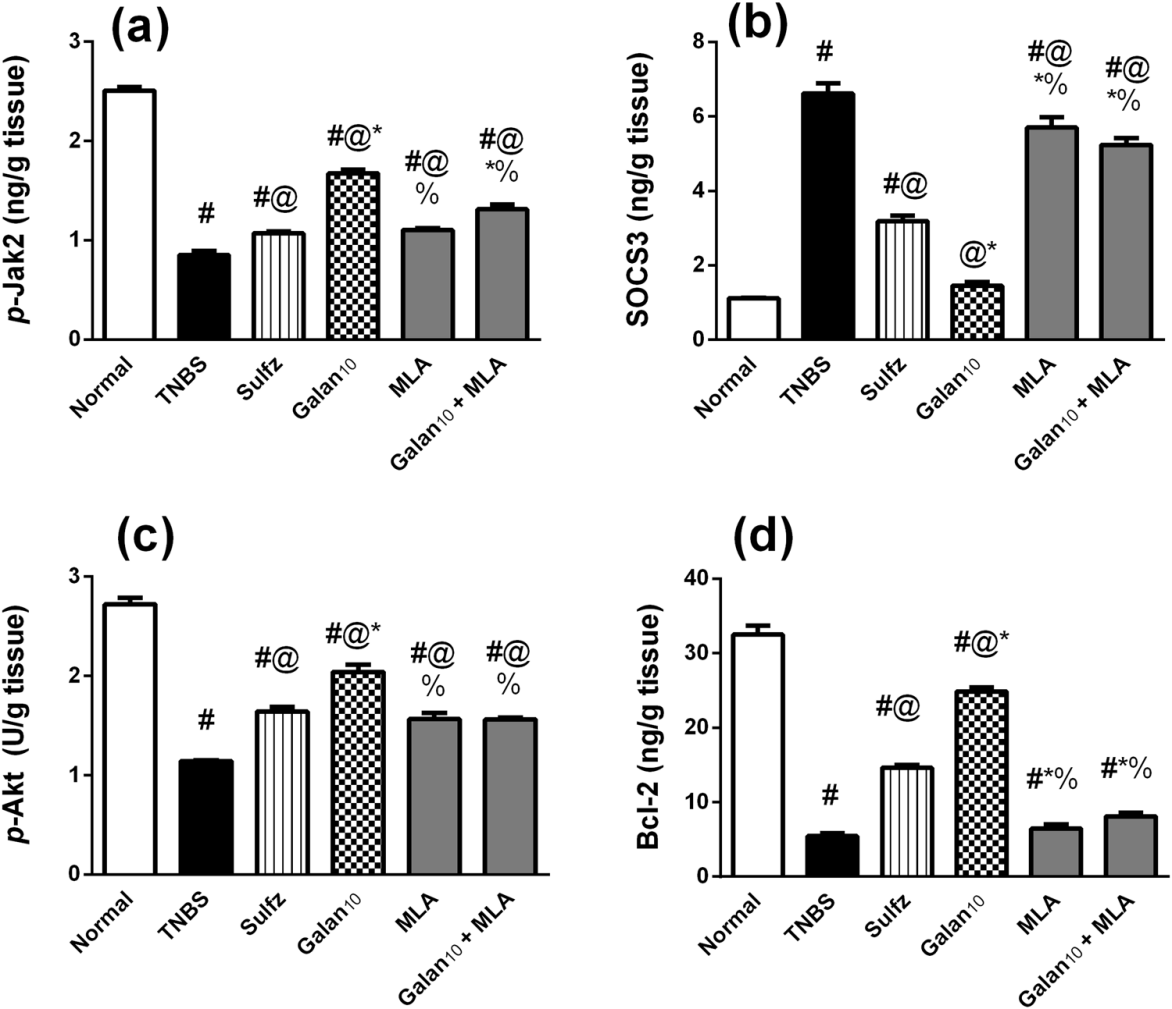
Effect of pretreatment with Galantamine (10 mg/kg; Galan_10_) alone and with methylcaconitine citrate (MLA, 5.6 mg/kg) on *p-*Jak2/SOCS3 signaling pathway; (**a**) p-Jak2 and (**b**) SOCS3 as well as the antiapoptotic proteins; (**c**) p-Akt and (**d**) Bcl-2 in rats with TNBS-induced colitis. Data are expressed as means ± S.E.M. Statistical analysis was performed using one-way ANOVA followed by Tukey’s Multiple Comparison; as compared to normal control (#),TNBS (@), Sulfz (*), and Galan_10_ (%).

The TNBS insult reduced the colonic content of *p-*Jak2 (Fig. 6a), while hightened that of SOCS3 (Fig. 6b). Pre-treatment with either Sulfz or Galan prevented the TNBS-induced alterations. Again, MLA blocked the effects of Galan on both *p*-Jak2 and SOCS3.

### Effect of Galan_10_ alone and with MLA on p-Akt and Bcl-2 in TNBS-induced colitis

TNBS-induced apoptosis was detected by the significant decrease in the colonic content of the anti-apoptotic parameters *p*-Akt and Bcl-2. These changes were largely prevented by the use of Galan and to a lesser extent with Sulfz. The Galan effects were curtailed upon using MLA, while the blocker alone showed no alteration in these parameters as compared to TNBS group. (Fig. 6c,d)

### Effect of different treatments on histological photomicrographs

**Figure 7:**
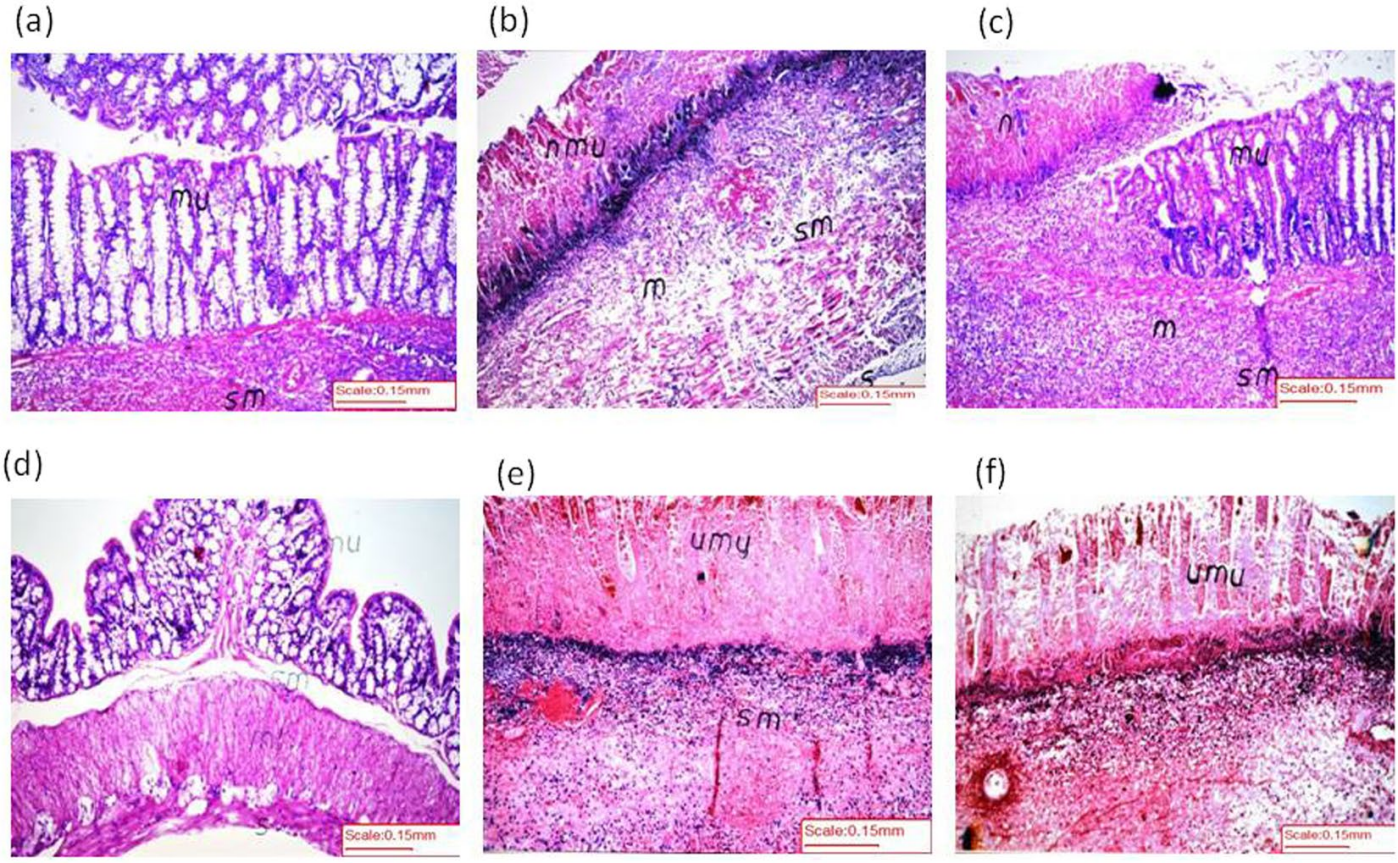
Effect of Galantamine (Galan) on histopathological changes of rat colon in TNBS model of colitis. (**a**) *Normal control rat:* normal histological structure of mucosa [*mu*] and submucosa [*sm*], (**b**) *TNBS control rat* showing wide area of ulceration, necrosis [*nmu*] and puss formation [*S*] in mucosal layer along with massive inflammatory cell infiltration [*m*] in submucosa [*sm*], (**c**) *Sulfasalazine pretreated rat* showing a focal area of necrosis [*n*] with ulceration and inflammatory cells infiltration [*m*] in mucosa and submucosa [*sm*], while the other adjacent wide area of the mucosa [*mu*] was intact, (**d**) *Galan (10 mg/kg) pretreated rat* showing normal histopathlogical structure of mucosa [*mu*], submucosa [*sm*], muscularis [*ml*] and serosa [*s*], (**e**) *Rat pretreated with methylcaconitine citrate (MLA*, *5*.*6 mg/kg)* showing necrosis, ulceration and hemorrhage in the mucosal layer [*umu*] with inflammatory reaction in the underlying submucosa [*sm*], (**f**) *Rat pretreated with Galan*_10_ *and MLA* showing focal necrosis, ulceration and hemorrhage in the mucosa [*umu*] with inflammatory reaction and suppuration in the underlying submucosa. (H&E staining, ×16 original magnification).

The histopathological results further confirmed the biochemical ones. Photomicrographs (Fig. 7) of (a) normal colon sections show normal colon structure and intact mucosal layer with the formation of crypts and global cells [*mu*], as well as the underlying submucosa [*sm*]. However, (b) TNBS reveals wide area of ulceration, necrosis [*nmu*] and puss formation [*S*] in mucosal layer along with massive inflammatory cell infiltration [*m*] in the submucosa [*sm*]. These modifications were substantially prevented by the pretreatment with (d) Galan_10_ and to a lesser extent with (c) Sulfz. The colon of animals pretreated with (e) MLA alone or (f) with Galan_10_ showed necrosis, ulcerations, and hemorrhage in the mucosal layer [*umu*] with inflammatory reaction in the underlying submucosa [*sm*].

## Discussion

The present study clearly indicates that Galan appraised its effect via activating the cholinergic anti-inflammatory pathway with the involvement of the Jak/STAT and NF-κB/HMGB-1/RAGE signaling pathways. The mechanisms are obviously orchestrated by the α7 nAChR, as blocking this receptor blunted all the beneficial effects of Galan. Pretreatment with Galan significantly reduced TNBS-induced gross damage and histopathological alterations along with a marked reduction in CMI.

These protective effects are endorsed by curbing neutrophil infiltration as proven herein and previously^25^. The inhibition of MPO is responsible in part for the blunted oxidative stress verified here by the reduced lipid peroxidation in Galan treated group. Neutrophils are linked to oxidative stress, which is a key factor in the propagation of mucosal damage in IBD^26^. Galan also abated the transcription factor *p*-NF-κB to mediate its anti-colitic effect and this synchronize with the findings of Neurath *et al*.^27^. This effect is mediated through α7 nAChR, which curtails the proteasomal degradation of inhibitory kappa B (IκB) to hinder the nuclear translocation of NF-κB^28,29^. MLA co-administration abolished the Galan effect to prove the role of the nicotinic receptor. Similarly, Sulfz also inhibited NF-κB to concur with a previous study^30^ reporting that Sulfz blocks the nuclear shifting of NF-κB by modulating IκBα and IκB kinase (IKK). Being a key regulator of several pro-inflammatory mediators, inhibition of NF-κB compels the decrease in TNF-α and ICAM-1 to hinder leukocyte transmigration with the consequent release/activation of MPO as reported here and earlier^30–32^. In a vicious cycle, however, TNF-α increases NF-κB through enhancing the IKKβ function and the ubiquitination of IκB^33^. Additionally, suppression of NF-κB causes a decrease in HMGB1and RAGE; the latter interacts with many ligands associated with inflammatory responses. To support the present results, Zen *et al*.^34^ showed that RAGE expression was up-regulated in colon tissue from IBD patients and experimentally after treatment with IFN γ and/or TNF-α. RAGE may play a dual role in the inflammatory response, where it activates NF-κB in a downstream cascade and may function as an adhesive receptor to be involved in inflammatory cell recruitment^35^. HMGB1 is one ligand of RAGE that mediates inflammation; thus, TNF- α, HMGB1, and NF-κB are involved in positive feedback loops that may help to sustain inflammation^36^. At the meantime, HMGB1 may signal *via* toll-like receptors to again activate inflammatory cascade and up-regulate leukocyte adhesion molecules^37^. Sulfz also abated the inflammatory mediators HMGB1, TNF-α and consequently RAGE, as compared to the colitis group. Indeed, Galan produced better results, where it normalized HMGB1 and TNF-α with a marked reduction in RAGE content. At the meantime, treatment with Galan together with MLA reverted the anti-inflammatory capacity of Galan to nail down the role of α7 nAChR.

Although both Sulfz and Galan produced similar reduction in NF-κB activity, however, the overall protective effect of Galan on colitis was more obvious than that of Sulfz implicating that other anti-inflammatory mechanisms or signaling pathways might play a role.

Another critical component of the cholinergic anti-inflammatory pathway appears to be mediated by the Jak2/STAT3 signaling in macrophages. This pathway is induced by either the binding of IL-6 or IL-10 to the cytokine receptors to mediate two different outcomes. In the present work, IL-10 was inhibited by TNBS leaving the effect of IL-6 to predominate, a cytokine known to be induced by TNBS^36,38^. Jak/STAT3 cue may be an upstream signaling for NF-κB activation, where stimulation of Jak/STAT3 is responsible partly for the generation of NF-κB^39^. The pathways of both STAT3 and NF-κB are thought to congregate to convey inflammatory signaling, as the NF-κB p65 homodimer recruits and associates with STAT3 upon activation^40,41^. Such inflammatory event is followed by the induction of SOCS3, which acts as a feedback inhibitor of such cascade mediated by IL-6, but not IL-10. In the present work, TNBS increased SOCS3, while inhibited the phosphorylated/active Jak2, pointing to the anti-inflammatory effect of SOCS3, as a compensatory response, at the time of determination. Moreover, increased SOCS3 has been reported to inhibit IL-6-induced Jak/STAT signaling, but not that initiated by IL-10^38^. The increased SOCS3 observed in the colitic animals is in line with the work of Suzuki *et al*.^42^, who detected increased SOCS3 expression in mouse models of IBD. Moreover, increased SOCS3 expression has been detected in inflamed biopsies of patients with UC compared with non-inflamed biopsies^43^.

In this study, Galan increased the anti-inflammatory cytokine IL-10, which may clarify in part the elevated phosphorylated/active Jak2 to mediate the anti-inflammatory effect of this pathway. Murray^38^ stated that although the Jak/STAT pathway activated by IL-10 is a process seemingly identical to that activated by IL-6, yet the downstream readouts are clearly diverse. Additionally, the stimulation of α7 nAChR on macrophages by Galan triggers the activation of its catalytic intracellular domain leading to the recruitment and phosphorylation of tyrosine kinase Jak2, along with the subsequent activation of the transcription factor STAT3^44^ to produce the anti-inflammatory effect. These authors found that activation of STAT3 signaling is critical for the anti-inflammatory effect of nicotine, which failed to reduce TNF-α production in cells that express mutated STAT3 in the phosphorylation domain, or the DNA-binding domain. STAT3 is generally recognized as an anti-inflammatory transcription factor contributing to the anti-inflammatory effects of IL-6 and IL-10^45,46^. However, STAT3 seems to exert its anti-inflammatory action indirectly, and does not directly inhibit the transcription of pro-inflammatory genes, such as TNF-α, IL-6 and iNOS^45,47^. In addition, STAT3 inhibits RAGE-mediated ICAM-1 upregulation and TNF-α generation^48^. Treatment with Galan, through inhibiting the release of pro-inflammatory cytokines and NF-κB, restored the balance between SOCS3 and *p*-Jak2, where SOCS3 was normalized in the present study, allowing Jak2/STAT3 signaling system to recover as evidenced by the increase in *p*-Jak2 towards normal levels. It is worth mentioning that increased IL-10, phosphorylation of Jak2, and restoration of SOCS3, caused by Galan were all inhibited by MLA.

In the present work, Galan also enhanced cell survival via increasing *p*-Akt and Bcl-2, effects that are also linked with the activation of Jak2 by Galan *via* the α7 nAChR. This cascade stimulates phosphatidyl-inositol-3-kinases, with the consequent production of phosphatidylinositol triphosphate^49^. The latter in turn phosphorylates/activates Akt to mediate several effects, such as suppressing apoptosis and up-regulating the anti-apoptotic Bcl-2 protein^50^. Again, the Galan-mediated increment of Akt and Bcl-2 was prevented by the concomitant treatment with MLA to pin down the pivotal role of the α7 nAChR in the anti-apoptotic effect of Galan.

In the current work, MLA alone did not alter the TNBS effect; the lacking effect of MLA on the severity of TNBS-induced colitis suggests that the basal levels of ACh in this IBD model are low and are not enough to be associated with any protective effects through the α7 nAChR. This could be explained on the basis that the intestinal muscularis resident macrophages expressing α7 nAChR are the ultimate target of the gastrointestinal cholinergic anti-inflammatory pathway^51^ and that TNBS inflammation down-regulates the expression of choline acetyltransferase in the muscularis externae^52^. However, stimulating this pathway with Galan increases ACh output through both the interaction of the vagus with the spleen (splenic pathway) and the cholinergic myenteric neurons in close contact with the muscularis macrophages. This was confirmed indirectly by the measurement of the plasma ACh level, where TNBS-induced colitis led to a reduction in plasma ACh level, but Galan pretreatment opposed it (supplement).

In conclusion, Galan has mediated its anti-colitic via positive allosteric modulation of the corner stone molecule α7 nAChR and through stimulating the cholinergic anti-inflammatory pathway that increased ACh to trigger a spectrum of anti-inflammatory effects. Galan has modulated a myriad of inflammatory/oxidant/apoptotic signals involving HMGB1/RAGE/NF-κB/TNF-α, ICAM-1/MPO, IL-10, Jak2/STAT3, and Akt/Bcl-2. Stimulation of the nicotinic α7 nAChR appears to be critical for the Galan effect, since its blockade by MLA abrogates almost all the beneficial anti-inflammatory signals.

## Electronic supplementary material

Supplementary Information
