# Supplementary material for: Galantamine anti-colitic effect: Role of alpha-7 nicotinic acetylcholine receptor in modulating Jak/STAT3, NF-κB/HMGB1/RAGE and p-AKT/Bcl-2 pathways
Source: Sci Rep. 2018 Mar 23;8:5110. doi: 10.1038/s41598-018-23359-6 (PMC5865178; doi:10.1038/s41598-018-23359-6)
Supplement: Supplementary file 1 — Supplementary Information [file 41598_2018_23359_MOESM1_ESM.pdf]

Galantamine anti-colitic effect: Role of alpha-7 nicotinic acetylcholine receptor in modulating Jak/STAT3, NF-κB/HMGB1/RAGE and *p*-AKT/Bcl-2 pathways

Shakeeb A. Wazea, Walaa Wadie*, Ashraf K. Bahgat, Hanan S. El-Abhar.

Department of Pharmacology & Toxicology, Cairo University, Cairo, Egypt

* corresponding: walaa.wadie@pharma.cu.edu.eg

The following figure illustrates the role of alpha-7 nicotinic acetylcholine receptor (α7 nAChR) in mediating the anti-colitic effect of galantamine (Galan) via Jak/STAT3, NF-κB/HMGB1/RAGE and p-AKT/Bcl-2 pathways.

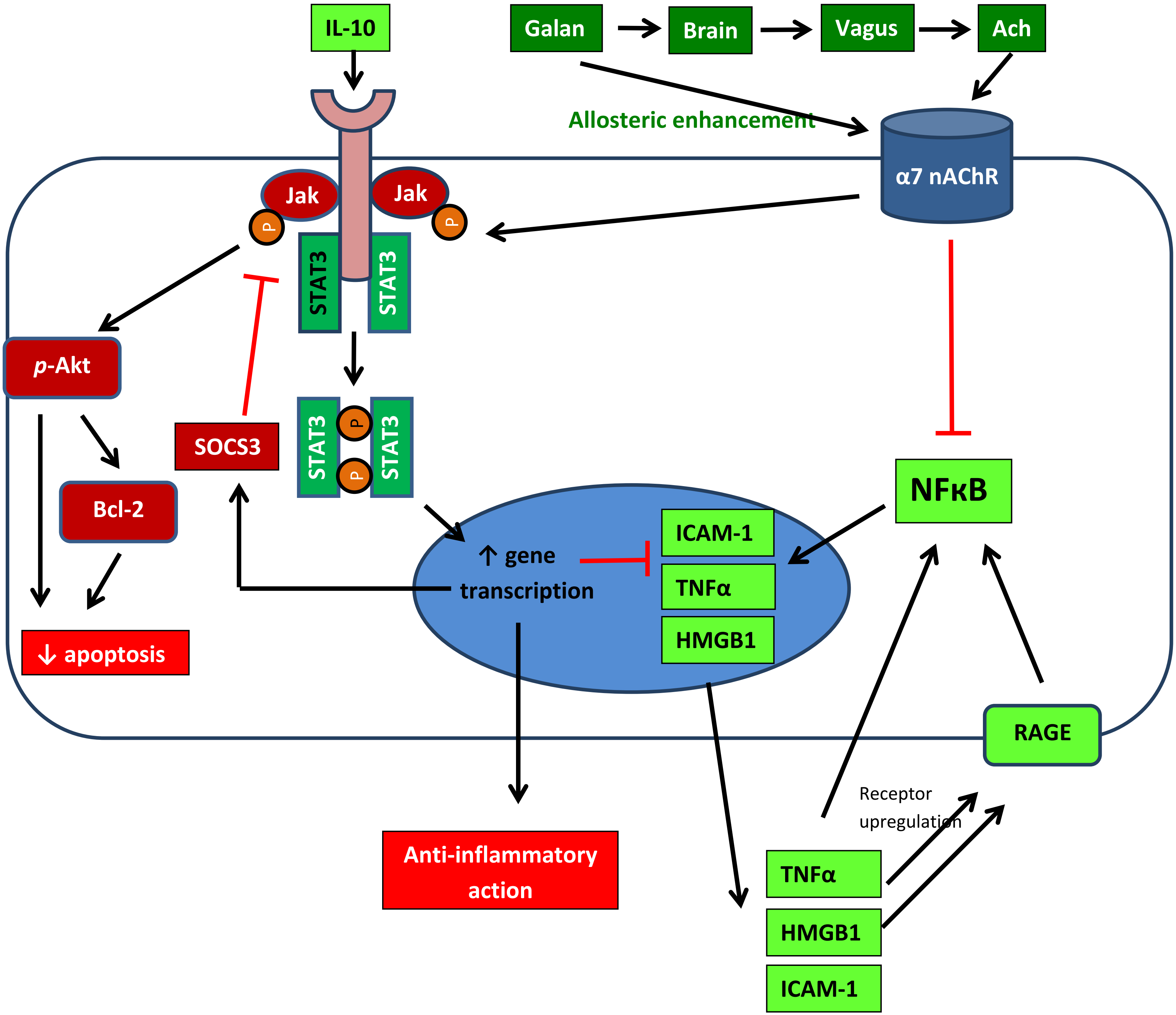
IL-10
Galan
Brain
Vagus
Ach
Allosteric enhancement
α7 nAChR
Jak
Jak
P
P
STAT3
STAT3
p-Akt
SOCS3
STAT3
STAT3
P
P
Bcl-2
NFκB
↑ gene transcription
ICAM-1
TNFα
HMGB1
↓ apoptosis
RAGE
Receptor upregulation
Anti-inflammatory action
TNFα
HMGB1
ICAM-1
